# Krüppel-Like Factor 6 Splice Variant 1: An Oncogenic Transcription Factor Involved in the Progression of Multiple Malignant Tumors

**DOI:** 10.3389/fcell.2021.661731

**Published:** 2021-03-18

**Authors:** Kang Hu, Qing-Kang Zheng, Rui-Jie Ma, Chao Ma, Zhi-Gang Sun, Nan Zhang

**Affiliations:** ^1^School of Clinical Medicine, Weifang Medical University, Weifang, China; ^2^Cheeloo College of Medicine, Shandong University, Jinan, China; ^3^Department of Thoracic Surgery, Jinan Central Hospital, Cheeloo College of Medicine, Shandong University, Jinan, China; ^4^Department of Oncology, Jinan Central Hospital, Cheeloo College of Medicine, Shandong University, Jinan, China

**Keywords:** KLF6, KLF6-SV1, proliferation, apoptosis, invasion, EMT

## Abstract

Krüppel-like factor 6 (KLF6) is one of the most studied members of the specificity protein/Krüppel-like factor (SP/KLF) transcription factor family. It has a typical zinc finger structure and plays a pivotal role in regulating the biological processes of cells. Recently, it has been considered to play a role in combatting cancer. Krüppel-like factor 6 splice variant 1 (KLF6-SV1), being one of the alternative KLF6 splicing isoforms, participates in tumor occurrence and development and has the potential to become a new target for molecular targeted therapy, although its action mechanism remains to be determined. The purpose of this article is to provide a comprehensive and systematic review of the important role of KLF6-SV1 in human malignant tumors to provide novel insights for oncotherapy.

## Introduction

### The KLF6 Gene

KLF6 is part of the Krüppel-like family and is a ubiquitously expressed nuclear transcriptional regulatory factor that is originally isolated and cloned from three independent tissues: placental cells (Koritschoner et al., 1997), hepatic stellate cells (Kim et al., 1998), and peripheral blood lymphocytes of B-cell chronic lymphocytic leukemia patients (El Rouby et al., 1997). KLF6 is located on human chromosome 10p15, which consists of 4 exons with an entire length of 7 kb, and it encodes a protein made up of 283 amino acids. KLF6 has an NH_2_ tag-end activation domain that is rich in proline and serine. Like other KLFs, KLF6 contains three COOH-terminal C_2_H_2_-type zinc-finger structures. These structures can be connected to the “GC box” and “CACCC box,” as well as to other promoter regions of the target gene. It can also regulate gene transcription through protein-protein interaction (Zhang et al., 1998; Song et al., 2003; Li et al., 2005).

**FIGURE 1:**
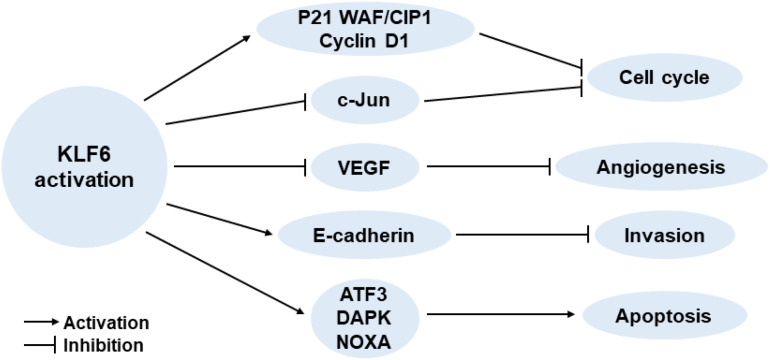
Effects of increased KLF6 expression on cancer-related key signaling pathways.

It has been reported that KLF6 serves important roles in regulating various cellular biological processes, including tissue growth and development (Fischer et al., 2001; Park et al., 2005), cell proliferation and differentiation (Inuzuka et al., 1999; Li et al., 2005), and vascular remodeling (Botella et al., 2002). Moreover, KLF6 is also increasingly appreciated for its tumor suppressor function. Although the exact mechanism of the anti-cancer effect of KLF6 remains elusive, some classic pathways have been described: activating p21 through p53-independent mode (Narla et al., 2001), reducing the cyclin D1/CDK4 complex through interaction with cyclin D1 (Benzeno et al., 2004), inhibiting c-Jun proto-oncoprotein activities (Slavin et al., 2004), decreasing vascular endothelial growth factor (VEGF) expression (DiFeo et al., 2006b), up-regulating E-cadherin (DiFeo et al., 2006a), and inducing apoptosis (Ito et al., 2004; Figure 1). Of course, there are still many unknown mechanisms of the tumor suppressor function of KLF6, which need to be further explored by researchers in the future.

### Alternative KLF6 Splicing in Cancer

**FIGURE 2:**
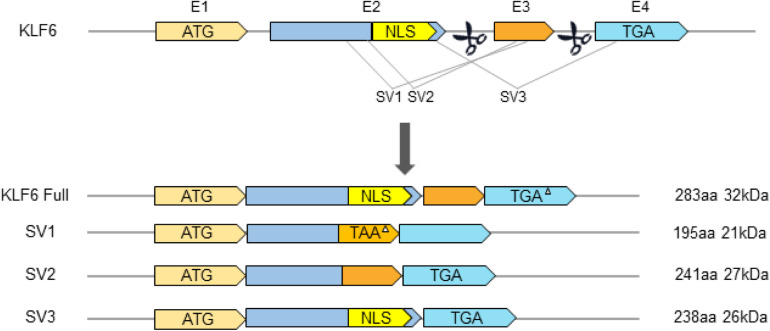
Schematic diagram of gene structure of KLF6 and its splicing variants. E1–E4 indicates exons 1–4. TGA or TAA represents translation Stop codons. NLS, Nuclear Localization Signal. The triangle (△) indicates that the Stop codon is in a different box from the KLF6 open reading box.

In recent years, with advances in the understanding of tumor pathogenesis, researchers have found that the inactivation of KLF6 plays an increasingly important role in promoting tumor production. There is considerable evidence that the inactivation of KLF6 by mutation or deletion is frequently observed in most human cancers. At the same time, KLF6 also often produces oncogenic splicing variants by increasing alternative splicing. Recently, Narla et al. (2005a) have found that single nucleotide polymorphism (SNP) of KLF6 can increase its alternative splicing, resulting in three splice variants SV1, SV2, and SV3 in prostate cancer patients. The wild-type KLF6 and SV3 variants are located in the nucleus because of the nuclear localization signal (NLS) in exon 2, while the SV1 and SV2 variants are mainly located in the cytoplasm due to the absence of NLS (Figure 2).

### The KLF6-SV1 Gene

Studies report that KLF6 can produce the oncogenic splicing variant KLF6-SV1 by increasing alternative splicing (Narla et al., 2005a; DiFeo et al., 2006b; Kremer-Tal et al., 2007). KLF6-SV1 is produced by alternative 5′ splice sites and is included in a novel 21 amino acid carboxy domain owing to out-of-frame splicing of exon 3. KLF6-SV1 loses three consecutive C_2_H_2_ zinc finger structures at the C-terminal but still retains the activation domain of the KLF transcription factor family that mediates biological activity between proteins. Abundant data indicate that wild-type KLF6 (WtKLF6) is a classical tumor suppressor and is frequently mutated in many cancers (Narla et al., 2001; Chen et al., 2003; Benzeno et al., 2004; Kremer-Tal et al., 2004). Among the three KLF6 splice variants, KLF6-SV1 is the most explicit in oncogenesis, and it can obviously antagonize the anti-cancer function of KLF6. KLF6-SV1 is often highly expressed in various human malignancies, which can significantly accelerate cell proliferation, invasion, and metastasis, resulting in a poor prognosis and low survival rate in cancer patients (Narla et al., 2005a,b).

## KLF6-SV1 Takes a Pivotal Role in Human Malignant Tumor Progression

### High KLF6-SV1 Expression Contributes to Cellular Proliferation and Survival

Although KLF6-SV1 exists in both normal and tumor cells, the expression of this cytoplasmic subtype is markedly up-regulated in a number of malignant tumors and is strongly associated with the proliferation of cancer cells. However, the molecular mechanism by which KLF6-SV1 is up-regulated and promotes cellular proliferation in human tumors remains to be identified.

It was found for the first time in 2004 that KLF6 expression in glioblastoma multiforme (GBM) can inhibit the growth and tumorigenicity of glioblastoma which could then activate the p21^Cip1/Waf1^ promoter both *in vitro* and *in vivo* (Kimmelman et al., 2004). A later study uncovered that increased KLF6-SV1 and reduced expression of KLF6 are frequent representations in GBM and the overexpression of KLF6-SV1 antagonize wild-type KLF6-mediated growth inhibition (Camacho-Vanegas et al., 2007). Using small interfering RNA (siRNA)-mediated gene targeting to silence KLF6-SV1 expression can reduce the cell proliferation of GBM cell lines, which indicates that KLF6-SV1 overexpression can promote the proliferation of GBM cell lines. Moreover, researchers found a similar phenomenon in prostate cancer. KLF6-SV1 overexpression significantly antagonized the ability of wild-type KLF6 (WtKLF6) to up-regulate p21 expression and inhibit cellular proliferation, which eventually led to increased cell proliferation while also significantly raising the risk of prostate cancer in males (Narla et al., 2005a).

**FIGURE 3:**
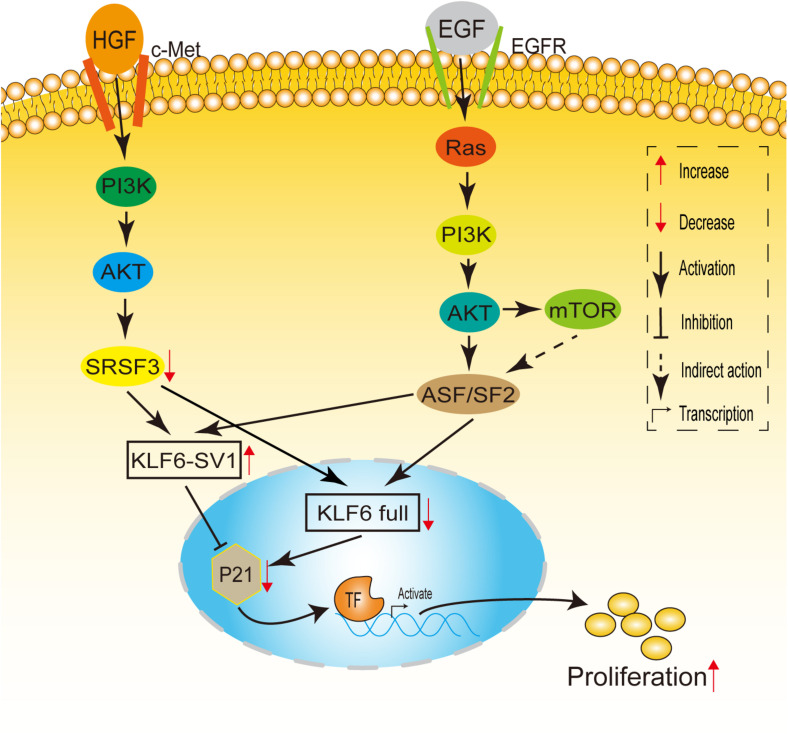
A model linking Ras and HGF signaling to KLF6 alternative splicing. HGF induces c-Met, PI3K/Akt, which downregulates SRSF1 levels. Decreased SRSF1 enhances KLF6 alternative splicing, altering the SV1/KLF6 full ratio, leading to increased cell proliferation. EGF receptor potentially can activate Ras signaling. When Ras is activated, signals are transduced through PI3-K/Akt to ASF/SF2, up-regulating KLF6 SV1 expression and leading to cellular proliferation. mTOR also may play a role in ASF/SF2-dependent or ASF/SF2-independent KLF6 alternative splicing.

Recently, one study showed that the expression of KLF6-SV1 is up-regulated not only in the carcinogenic Ras/PI3K/Akt-dependent signal pathway (Yea et al., 2008), but also in the carcinogenic HGF/PI3K/Akt-dependent signal pathway (Munoz et al., 2012), thereby changing the relative ratio of KLF6 Full to KLF6-SV1, which in turn leads to increased proliferation of liver cancer cells (Figure 3). Through further studies on mouse models and samples of hepatocellular carcinoma (HCC) patients infected with HCV, it was found that the inactivation of KLF6 and the increase of KLF6-SV1 have separate and complementary effects on tumor propensity. Hepatocyte-specific deletion of KLF6 promoted the enhancement of proliferation, while KLF6-SV1 overexpression largely caused the increase in tumor grade (Vetter et al., 2012). Moreover, Zhang et al. (2019) found that KLF6-SV1 can regulate the PI3K-AKT signaling pathway and the Bcl-2/Bax axis in non-small cell lung carcinoma (NSCLC), resulting in promoting tumor cell proliferation.

Although the up-regulation of KLF6-SV1 tends to promote tumor cell proliferation in most malignant tumors, its effect on cells in breast cancer is an exception. In breast epithelial and cancer lines, KLF6-SV1 overexpression was identified to have a cell proliferation–independent effect in both *in vitro* and *in vivo* models of breast cancer. Hatami et al. (2013) observed that the expression of KL6-SV1 mainly sustained breast cancer cell survival through the evasion of apoptosis, but not proliferation. However, the specific mechanism is still unclear. Further prospective studies in independent large well-annotated clinical cohorts are necessary to validate these initial observations.

### KLF6-SV1 Provides Tumor Cells With Growth and Survival Advantage by Inhibiting Apoptosis

**FIGURE 4:**
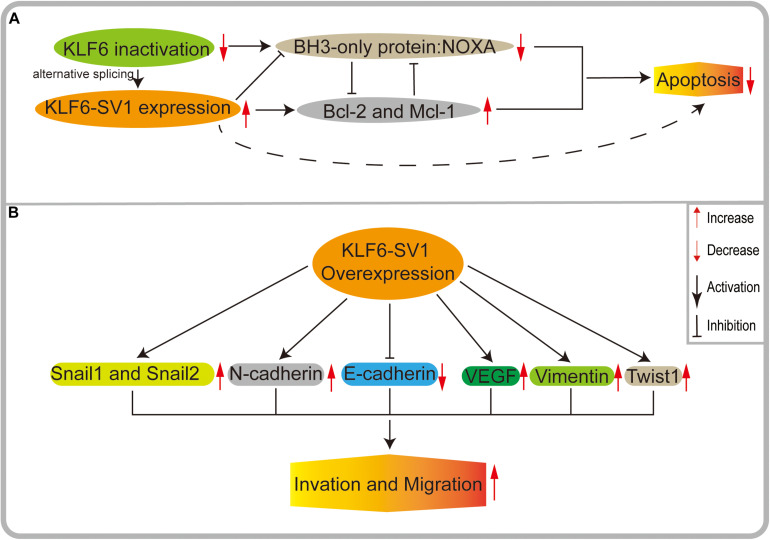
**(A)** KLF6 produces oncogenic splicing KLF6-SV1 by increasing alternative splicing. KLF6-SV1 can bind to the pro-apoptotic BH3-only protein NOXA, resulting in the degradation of their mutual HDM2-dependent and accelerating the death of NOXA. High expression of KLF6-SV1 can increase the intracellular concentration of anti-apoptotic proteins McL-1 and BCL-2 and effectively prevent the apoptosis of cancer cells. **(B)** In KLF6-SV1 overexpressed tumor cells, E-cadherin expression is significantly decreased, while the expressions of VEGF, Twist1, N-cadherin, Vimentin, Snail1, and Snail2 are significantly increased, finally leading to tumor invasion and metastasis.

Recently, many scholars have proposed that KLF6-SV1 may play a significant role in regulating apoptosis (Figure 4A), but its potential mechanism remains largely undefined. There is an abundance of correlative evidence indicating that KLF6-SV1 is an anti-apoptotic protein and is not correlated with the p53 status of tumor cells. KLF6-SV1 can bind to the pro-apoptotic protein Noxa and degrade it, thereby providing survival advantages for tumor cells by inhibiting apoptosis. In the prostate cancer model, Narla et al. (2008) found that the number of apoptotic cells in KLF6-SV1-overexpressing tumors was 70% less than that in the control group. At the same time, KLF6-SV1 overexpression has also been shown to significantly promote the survival of lung cancer cell lines, which may be related to the increased expression of the pro-antiapoptotic gene Bcl-2 and the inhibition of the expression of the pro-apoptotic gene Noxa (DiFeo et al., 2008; Figure 4A). In chemotherapy-resistant lung adenocarcinoma cells, the expression level of KLF6-SV1 was significantly increased. KLF6-SV1 overexpression is likely to block cisplatin chemotherapy by up-regulating the expression of Mcl-1 and reducing the expression of Noxa. Cancer cells become resistant to cisplatin-induced apoptosis, and the anti-cancer effects of cisplatin are reduced when the apoptotic pathways are blocked (DiFeo et al., 2008; Sangodkar et al., 2009). Researchers have also made new advances in ovarian cancer models. KLF6-SV1 can bind to the pro-apoptotic BH3-only protein Noxa, resulting in the degradation of their mutual HDM2-dependence and also accelerating the death of apoptosis protein BH3. Meanwhile, it can increase the intracellular concentration of Mcl-1 and effectively prevent the apoptosis of cancer cells (DiFeo et al., 2009a; Figure 4A). Kokhaei et al. (2018) identified that KLF6-SV1 may be involved in the anti-apoptotic effect of T cells on chronic lymphoblastic leukemia cells both *in vivo* and *in vitro*, which leads to the deterioration of chronic lymphoblastic leukemia patients.

Based on increasing experimental evidence, there is reason to believe that KLF6-SV1 overexpression inhibits apoptosis and provides tumor cells with a growth and survival advantage in malignant tumors and is accompanied by chemotherapy resistance in some cancer cells. However, its specific anti-apoptotic mechanism remains to be further explored.

### KLF6-SV1 Enhances Invasion and Migration

As an inhibitory factor of cell invasion, E-cadherin plays critical roles in the occurrence and development of cancers, and its stable expression is the premise of maintaining the normal biological functioning of cells. The decrease of E-cadherin expression is a critical molecular event contributing to dysfunctional cell adhesion, inducing tumor invasion and metastasis (Naora and Montell, 2005; Figure 4B). DiFeo et al. found that a low expression of KLF6 and a high expression of KLF6-SV1 inhibited the activation of the E-cadherin gene promoter and significantly decreased its expression in ovarian cancer cells. Their results showed that the action of KLF6 and KLF6-SV1 on E-cadherin could affect tumor invasion (DiFeo et al., 2006a). Subsequently, one study has shown that KLF6 low expression and KLF6-SV1 high expression can significantly increase the vascular endothelial growth factor (VEGF) *in vivo*, thus enhancing the invasiveness of cells. At the same time, it was found that a higher expression of KLF6-SV1 was related to more aggressive ovarian cancers and may be specific to histological-type cancers (DiFeo et al., 2006b).

Soon afterward, through a study of the biopsy specimens of 50 patients with primary nasopharyngeal carcinoma (NPC) who did not receive any treatment, researchers also identified that high KLF6-SV1 expression may result in E-cadherin decreasing significantly. Additionally, the level of KLF6-SV1 was positively correlated with young patients, indicating that this oncogenic variant can be favorable to enhance the aggressiveness of the juvenile form of NPC (Debouki-Joudi et al., 2018). The negative correlation between the KLF6-SV1 and E-cadherin expression shows that the oncogenic variant could promote NPC invasion and metastasis. However, the specific mechanism of KLF6-SV1 in NPC needs to be further studied.

In the mouse model of prostate cancer, high expression of KLF6-SV1 can promote tumor cell proliferation and angiogenesis, and likewise reduce apoptosis, thereby mediating tumor invasion, migration, and spread (Ashkenazi and Dixit, 1998; DiFeo et al., 2008). Further study revealed that KLF6 expression decreased significantly in prostate cancer metastases but that KLF6-SV1 expression increased, especially in hormone refractory prostate cancer metastases. Studies also showed faster lymph node, bone, and brain metastasis, but these did not affect the growth of primary tumors. Thus, it can be seen that the expression levels of KLF6-SV1 are a significant index by which the invasion and migration of prostate cancer can be judged (Narla et al., 2008).

### KLF6-SV1 Alters the Expression Levels of EMT-Related Genes

Epithelial-mesenchymal transition (EMT) takes a pivotal role in many biological processes. EMT can promote tumor cell invasion and metastasis by changing cell-cell interactions and cell-matrix interactions (Inoue et al., 2015). EMT is implemented by EMT-activating transcription factors (EMT-TFs), including the ZEB, TWIST, and SNAIL families. In the course of studying the mechanism of KLF6-SV1 and tumor migration, we unintentionally found that KLF6-SV1 is closely related to a variety of EMT-activated transcription factors, and that the effect of KLF6-SV1 on tumor invasion and migration is likely to be mediated by the regulation of EMT (Figure 4B).

The transcription factor TWIST1 plays a key role in EMT, tumor development, and metastasis. In recent years, it has been found that the expression of TWIST1 is closely related to high-grade invasive breast cancer (Thisse et al., 1987; Yang et al., 2004; Mironchik et al., 2005; Ansieau et al., 2008). Hatami et al. found that KLF6-SV1 overexpression led to a significant up-regulation of the TWIST1 protein in a cohort of 671 lymph node-negative breast tumor patients. Their data also demonstrated that KLF6-SV1 was regulated by TWIST1 and was an early driver/initiator of breast cancer progression and metastasis (Hatami et al., 2013). Another study showed that high KLF6-SV1 expression promoted M2 macrophages’ infiltration by regulating the expression level of TWIST1 and CCL2, which eventually led to the involvement of EMT in the invasion and metastasis of lung cancer cell lines (Wang et al., 2019).

With further studies of lung cancer, researchers found that E-cadherin expression was reduced in clones of SK-MES-1 cells upregulated by KLF6-SV1, while SNAIL1, SNAIL2, Vimentin, and N-cadherin expression were significantly increased. On the contrary, after transfection of KLF6-SV1 siRNA into A549 cells, the expression of E-cadherin was up-regulated, while the expressions of N-cadherin, Vimentin, SNAIL1, and SNAIL2 were down-regulated, which further proves that KLF6-SV1 may mediate the occurrence and development of related tumors by regulating EMT activation transcription factors (Zhang et al., 2019).

### KLF6-SV1 Expression Is Increased in Some Cancer Samples and Correlates With Poor Survival

The high expression of KLF6-SV1 has been shown to correlate with poor survival in patients with prostate cancer (Narla et al., 2008), ovarian cancer (DiFeo et al., 2006b), lung cancer (DiFeo et al., 2008; Zhang et al., 2018), pancreatic cancer (Hartel et al., 2008), and hepatocellular carcinoma (Vetter et al., 2012).

DiFeo et al. (2008) detected the expression of KLF6-SV1 at the mRNA level in a total of 70 patients with lung adenocarcinoma by using PCR, and their data indicated that KLF6-SV1 was overexpressed in lung adenocarcinoma and was related to poor survival rates in adenocarcinoma patients. Moreover, in a clinical comparative study of 79 patients with non-small cell lung carcinoma (NSCLC), researchers found that KLF6-SV1 expression levels in lung adenocarcinoma were dramatically higher than those in squamous cell carcinoma, and the high expression of KLF6-SV1 was closely associated with lymph node metastasis, tumor stage, and poor prognosis of NSCLC patients, so they postulated that KLF6-SV1 could be used as a new prognostic biomarker after surgery (Zhang et al., 2018).

We further found that in addition to lung cancer, KLF6-SV1 overexpression in some other malignant tumors is closely correlated to the prognosis of patients. In prostate cancer, patients with high KLF6-SV1 expression had a median survival of nearly 30 months, compared to 80 months in low KLF6-SV1 expression patients (Narla et al., 2008). Researchers also found that KLF6-SV1 expression increased significantly in pancreatic cancer tissues, that the expression of KLF6-SV1 increased as much as 5 times with tumor progression, and that its increase was significantly related to tumor stage and survival rate (Hartel et al., 2008). Similarly, in liver cancer patients, the increasing KLF6-SV1/KLF6 ratio in tumor tissue was related to more advanced disease features (Vetter et al., 2012).

## Targeted KLF6-SV1 Reduction Leads to Decreased Tumor Growth and Spontaneous Apoptosis

Evidence has demonstrated that KLF6 and KLF6-SV1 serve key roles in the malignant progression of various tumor types; specifically, the high expression of KLF6-SV1 is closely related to cell proliferation, tumor apoptosis, invasion, and migration. Moreover, KLF6-SV1 has been considered a new anti-tumor target and is of high importance to the control of apoptosis independently of p53 function in tumor cells, which suggests its potential as a biological target of anti-tumor therapy.

In recent years, KLF6-SV1-related targeted therapy has made new progress under the unremitting exploration of scientific researchers. The siRNA-mediated inhibition of KLF6-SV1 could significantly reduce the proliferation of the glioma cell line (Camacho-Vanegas et al., 2007; Tchirkov et al., 2010). It was also discovered that the siRNA-mediated inhibition of KLF6-SV1 could lead to spontaneous apoptosis of prostate cancer cells and inhibit tumor growth (Ashkenazi and Dixit, 1998; Narla et al., 2008). KLF6-SV1 siRNA transfection can also inhibit the proliferation and invasion of gastric cancer cells (Chen et al., 2011). In lung adenocarcinoma cells, the up-regulation of KLF6-SV1 can reduce the sensitivity of patients to cisplatin chemotherapy, while the down-regulation of KLF6-SV1 mediated by siRNA can restore the sensitivity of lung adenocarcinoma cells to chemotherapy (DiFeo et al., 2008; Sangodkar et al., 2009). Moreover, a previous study showed that down-regulating the level of KLF6-SV1 by siRNA can significantly reduce the proliferation and invasion of ovarian cancer cell line SKOV-3 and also restore the sensitivity of ovarian cancer cells to cisplatin (DiFeo et al., 2009a,c). Therefore, if siRNA-mediated inhibition of KLF6-SV1 and cisplatin chemotherapy are used in combination, the superimposed therapeutic effect of apoptosis recovery induced by them can be significantly higher than that with either agent alone. This process will bring unlimited possibilities for future treatments of patients with malignant tumors.

Currently, a substantial body of literature has highlighted the potential effectiveness and specificity of future siRNA/RNAi-based therapies in malignant tumors (Elbashir et al., 2001; Gewirtz, 2007; DiFeo et al., 2009b; Weng et al., 2019). That is, on the one hand, siRNA-mediated inhibition of KLF6-SV1can up-regulate the pro-apoptotic protein Noxa and reduce anti-apoptotic proteins, thus accelerating the apoptosis of tumor cells. On the other hand, it can indirectly block the invasion and migration of tumors so as to inhibit tumor growth and improve the prognosis of patients.

## Conclusion and Future Prospects

As one of the alternative splice variants of KLF6, KLF6-SV1 is specifically up-regulated in multiple malignant tumors. The production process of KLF6-SV1 is a multi-factor and multi-mechanism process, one that can regulate the occurrence and development of tumors in many ways. Although current studies on KLF6-SV1 have not had a significant impact on the practice of tumor treatment and prognosis, researchers are still making efforts.

The first hot topic in academia is KLF6-SV1-associated tumor cell proliferation, invasion, metastasis, and prognosis. A considerable number of small-sample studies are involved in a variety of human malignant tumors, such as prostate cancer (Narla et al., 2008, 2005a,b), lung cancer (DiFeo et al., 2008; Sangodkar et al., 2009; Zhang et al., 2018, 2019; Wang et al., 2019), breast cancer (Hatami et al., 2013), ovarian cancer (DiFeo et al., 2006b, 2009a,c), gastric cancer (Chen et al., 2011), etc. (Table 1). However, more large-sample experiments are needed to further understand the specific molecular mechanisms and pathways of KLF6-SV1 regulation in malignant tumors.

**TABLE 1:** The regulatory role of KLF6-SV1 overexpression on tumor-related molecules.

Tumor type	Molecules	Key results	References
Prostate cancer	P21, Noxa, Bcl-2, caspase-3, caspase-8, c-myc, MMP9	In KLF6-SV1-overexpressing cell lines, the expression of the oncogene c-myc and anti-apoptotic Bcl-2 was increased, with concomitant reduction of the cyclin-dependent inhibitor p21 and Noxa, thereby leading to cell proliferation and apoptosis. Increased invasion in KLF6-SV1 cell lines was associated with increased expression of MMP9.	Narla et al., 2005a,b, 2008
Lung cancer	P21, Noxa, Mcl-1, Bcl-2, Bax, MMP9,cadherin, N-cadherin, Vimentin, SNAIL1, SNAIL2, TWIST1	High levels of KLF6-SV1 and anti-apoptotic protein and decreased expression of pro-apoptotic protein result in increased cellular proliferation and cell survival. KLF6-SV1 expression is significantly associated with pN and pTNM stage and poor survival in NSCLC patient.	DiFeo et al., 2008; Sangodkar et al., 2009; Zhang et al., 2018, 2019; Wang et al., 2019
Breast cancer	TWIST1, E-cadherin, N-cadherin	KLF6-SV1 drives breast cancer metastasis and is associated with poor survival.	Hatami et al., 2013
Ovarian cancer	P21, Noxa, Bcl-2, Mcl-1, E-cadherin, VEGF	KLF6-SV1 up-regulation resulted in increased proliferation, invasion, angiogenesis, and tumorigenicity.	DiFeo et al., 2006b, 2009a,c
Gastric cancer	P21, Noxa, Bad, Bim, Bak, Bcl-xl, Mcl-1, Bcl-2, caspase-9, Keratin4, E-cadherin, MMP9, uPA, VEGF	KLF6-SV1 overexpression mediate cell proliferation/survival, angiogenesis, motility and invation.	Chen et al., 2011

Secondly, anti-tumor research related to KLF6-SV1 has also made gratifying progress. The intervention of KLF6-SV1 may be a potential new therapeutic strategy for the treatment of human malignant tumors. SiRNA-targeted inhibition and down-regulation of KLF6-SV1 expression can lead to long-term silencing of tumor cells, thereby inhibiting tumor growth and obviously improving the prognosis of malignant tumor patients (DiFeo et al., 2009b). Thus, the horizon for the use of siRNA/RNAi seems to beckon with the potential for a “pharmacopeia of tomorrow” for a lot of human diseases (Gewirtz, 2007). However, the number of target genes used in siRNA therapy is limited, and there are many obstacles including the specificity of target genes and the delivery efficiency of siRNAs. In the next few years, further research is needed to determine more specific targets and develop more effective delivery systems for siRNA therapy.

Currently, there are few animal experiments on KLF6-SV1, and most animal experiments have adopted xenograft models. This experimental method is time-consuming, low in accuracy, and lacks the microenvironment for human tumor growth. Although xenograft models can faithfully recapitulate many aspects of tumorigenesis, ultimately, the true potential of KLF6-SV1 to drive tumor development needs to be tested in disease-relevant transgenic animal models of the disease. Transgenic animal model has the characteristics of replicability, controllability and short experimental period, which is more conducive to understand mechanisms of disease and explore potential interventional strategies. Therefore, with our ever-expanding knowledge of the exact relationship between KLF6-SV1 and human cancer, it is urgent to apply these strategies first to transgenic animal models and then to clinical studies. We are confident that, ultimately, developing a successful therapeutic approach to regulate KLF6-SV1 will play a key role in treating numerous therapy-resistant and metastatic tumors. Moreover, further knowledge on molecular mechanisms regulated by KLF6-SV1 would provide important insight concerning the regulation of significant pro-oncogenic pathways. Such knowledge would also provide potential tumor-related specific targets for cancer treatment.

In short, there is still plenty of work that needs to be done in studying the role of KLF6-SV1 in malignant tumors progression and in understanding how to make it benefit patients in the future.

## Author Contributions

Z-GS and NZ designed the work. KH wrote the manuscript. Q-KZ and R-JM prepared the figures and tables. CM drafted and revised the manuscript. All the authors contributed to manuscript revision, read and approved the submitted version.

## Conflict of Interest

The authors declare that the research was conducted in the absence of any commercial or financial relationships that could be construed as a potential conflict of interest.
